# Mild to Severe Neurological Manifestations of COVID-19: Cases Reports

**DOI:** 10.3390/ijerph18073673

**Published:** 2021-04-01

**Authors:** Gabriele Melegari, Veronica Rivi, Gabriele Zelent, Vincenzo Nasillo, Elena De Santis, Alessandra Melegari, Claudia Bevilacqua, Michele Zoli, Stefano Meletti, Alberto Barbieri

**Affiliations:** 1 Anaesthesia and Intensive Care, Azienda Ospedaliero-Universitaria di Modena, 41125 Modena, Italy; 2 Department of Biomedical, Metabolic and Neural Sciences, Neuroscience Post Graduate School, University of Modena and Reggio Emilia, 41125 Modena, Italy; veronica.rivi@unimore.it (V.R.); michele.zoli@unimore.it (M.Z.); 3 Neuroradiology, Azienda Ospedaliero-Universitaria di Modena, 41125 Modena, Italy; gabriele.zelent@gmail.com; 4 Department of Laboratory Medicine, Azienda Unità Sanitaria Locale, 41125 Modena, Italy; vincenzo.nasillo@unimore.it (V.N.); e.desantis@ausl.mo.it (E.D.S.); a.melegari@ausl.mo.it (A.M.); 5 School of Anaesthesia and Intensive Care, University of Modena and Reggio Emilia, 41125 Modena, Italy; clausbevilacqua@gmail.com (C.B.); alberto.barbieri@unimore.it (A.B.); 6 Neurology, Azienda Ospedaliera Universitaria di Modena, 41125 Modena, Italy; stefano.meletti@unimore.it

**Keywords:** COVID-19 outbreak, anosmia, encephalitis thrombosis, intensive care unit

## Abstract

The main focus of Coronavirus disease 2019 (COVID-19) infection is pulmonary complications through virus-related neurological manifestations, ranging from mild to severe, such as encephalitis, cerebral thrombosis, neurocognitive (dementia-like) syndrome, and delirium. The hospital screening procedures for quickly recognizing neurological manifestations of COVID-19 are often complicated by other coexisting symptoms and can be obscured by the deep sedation procedures required for critically ill patients. Here, we present two different case-reports of COVID-19 patients, describing neurological complications, diagnostic imaging such as olfactory bulb damage (a mild and unclear underestimated complication) and a severe and sudden thrombotic stroke complicated with hemorrhage with a low-level cytokine storm and respiratory symptom resolution. We discuss the possible mechanisms of virus entrance, together with the causes of COVID-19-related encephalitis, olfactory bulb damage, ischemic stroke, and intracranial hemorrhage.

## 1. Introduction

During the early phases of the Coronavirus disease 2019 (COVID-19) pandemic, the healthcare workforce was focused on treating the high number of patients with severe respiratory complications admitted to hospitals. However, it did not take long for physicians to understand that severe respiratory illness was only one of the clinical manifestations of COVID-19. A growing number of patients with symptoms, such as anosmia and dysgeusia, sometimes occurring in the absence of other clinical features, were diagnosed with COVID-19.

Acute cerebrovascular disease also emerged as an important complication caused directly or indirectly by the novel severe acute respiratory syndrome coronavirus 2 (SARS-CoV-2). As the COVID-19 pandemic progressed, reports of neurological manifestations, including encephalitis, cerebral thrombosis, neurocognitive (dementia-like) syndrome, or delirium, significantly increased. At the same time, recognizing the neurological disease in patients whose respiratory infection was mild or asymptomatic became extremely challenging [1,2,3]. The rate of patients admitted to intensive care units (ICUs) for COVID-19 neurological manifestations dropped over time; however, these patients might be left with severe neurological sequelae.

The neurological complications of SARS-CoV-2 present many similarities with those described in 2003 for severe acute respiratory syndrome (SARS) and in 2012 for the Middle East acute respiratory syndrome (MERS), suggesting that human respiratory Coronaviridae act through similar neuroinvasive and neurotrophic mechanisms. Coronaviruses are molecularly related in structure and mode of replication. By this, many researchers worldwide are focusing on understanding the mechanisms responsible for COVID-19 neurologic involvement.

The crucial pathophysiology behind the respiratory failure may be due to central nervous system (CNS) pathology [4,5]. Most neurological complications are currently thought to be related to the systemic effects of the viral infection, including cytokine release, immune-mediated inflammatory syndromes, and hypercoagulability [6].

Here, we present two different case reports of patients with neurological symptoms from COVID-19. The first patient was chosen for the neurological damage still present 30 days after the infection; whereas the second case showed a sudden unusual cerebral thrombosis complicated with acute hemorrhage after the cytokine storm decreased. We also discuss the putative neuro-disease mechanisms by which COVID-19 acts.

## 2. Materials and Methods

We selected two patients with uncommon neurological complications of COVID-19 infection. The first patient was chosen for the neurological damage still present 30 days after the infection. Although COVID-19 is known to directly damage olfactory sensory cells, there is limited literature consisting of case reports or series on olfactory bulb imaging in COVID-19 olfactory dysfunction [7]. The second case was chosen for the sudden neurological complications in a patient with a low level of cytokine storm, as research has demonstrated that pro-inflammatory cytokines are critically involved in abnormal clot formation and platelet hyperactivation [8]. Then, we discussed the possible mechanisms of the virus entrance together with the causes of COVID-19-related encephalitis, ischemic stroke, and intracranial hemorrhage. We also included a brief updated review of the literature to illustrate what is known about COVID-19 more broadly, bearing in mind that it is a highly complex syndrome with systemic effects and multiple organ damages.

Early reports indicated that patients with COVID-19 may have neurological manifestations and that preexisting neurological morbidities may result in poorer outcomes. Understanding the potential neurological and cerebrovascular manifestations in COVID-19 cases is crucial for appropriate diagnoses and treatments. We discussed the cases and investigated the potential pathophysiological mechanisms of neurological manifestations of the COVID-19 pandemic.

## 3. Case Report 1

A 31-year-old female patient reported anosmia and ageusia at the beginning of the COVID-19 pandemic in Europe and America (March 2020). The patient was previously healthy and had no relevant medical history. She was tested for SARS-CoV-2 three times: the first laboratory test for COVID-19 infection had an inconclusive value, the second was negative, whereas the third test, performed 20 days after the onset of the symptoms confirmed the previous infection of COVID-19 with positive IgG, and negative IgM, consistently with what is reported by recent literature.

**Figure 1:**
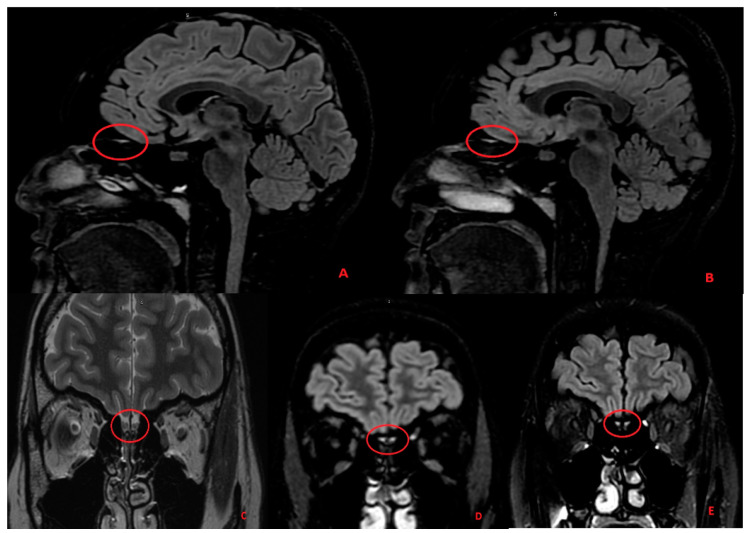
In the images above, we can see the hyperintensity of both olfactory bulbs (Panel **A**,**B**, sagittal FLAIR images) and olfactory tracts (Panel **C** coronal T2 FRFSE image; Panel **D**,**E** coronal FLAIR images) in the sequences obtained in the first magnetic resonance (MR) executed on the patient. In the coronal T2 FRFSE sequence (Panel **C**), we can also see that both olfactory tracts are swollen and hyperintense in their central portion, and that is even more evident for the right olfactory tract.

As these symptoms were still persistent 25 days after their onset, we performed further exams, including magnetic resonance (MR) and serum antibody analysis. Cerebrum spinal fluid was not investigated for the late diagnosis. The laboratory values were significantly altered for antibodies against myelin-associated glycoprotein (MAG), a marker of Schwann cell damage, and N-methyl-D-aspartate (NMDA) receptor antibodies, which is a marker of encephalitis. The MR revealed a persistent hyperintensity on the olfactory nucleus, a typical sign of encephalitis with the negative antibodies test (Figure 1).

According to the otorhinolaryngology examination, the patient did not experience nasal obstruction, and no abnormalities were observed in her nasopharyngeal mucosa during the medical examination. Then, to promote the recovery of both the senses, the patient began an empirical therapy with vitamin C integrators. Vitamin C, given its anti-inflammatory and immunomodulating properties, represents a potential therapeutic candidate both for the prevention and amelioration of COVID-19 infections and as an adjunctive therapy in the critical care of COVID-19 [9].

The patient chose not to perform a further MR for worsening claustrophobia after the brain MR. Ninety days after the onset of the symptoms, the patient noticed a small improvement in her sense of taste, but the anosmia was still partially present. No other neurological symptoms were present or concomitant.

### 3.1. Discussion Part I—Neuro-Invasion Mechanism: Can SARS-CoV-2 Directly Invade the CNS?

While initially fever, cough, and dyspnea were thought to be the principal symptoms of COVID-19, other unusual symptoms, such as altered olfactory function have been increasingly recognized. Isolated anosmia and hyposmia have been reported as a marker of COVID-19, and its onset often is concomitant with a decrease in taste sensation. The study of these symptoms is fundamental to elucidate the mechanisms by which SARS-CoV-2 invades the Cerebral Nervous System (CNS). The neuro-invasive property has been demonstrated as a common feature of human coronavirus (HCoV) diseases [10].

The exact mechanism of HCoVs entering the CNS is still not reported; however, different transmission routes have been suggested. To be neuroinvasive, HCoVs and SARS-CoVs may enter both from the periphery and directly by infecting the blood–brain barrier (BBB)’s endothelial cells. Having entered the blood, the virus can either remain free for a period before it can infect the endothelial cells of the BBB or infect leukocytes, which will become a viral reservoir for dissemination to other sites [11].

The entry of COVID-19 into human host cells appears to be primarily mediated by a cellular receptor, i.e., angiotensin-converting enzyme 2 (ACE2) [12]. ACE2 is expressed in human airway epithelia, lung parenchyma, vascular endothelial cells, the kidneys, the small intestines, and the brain. Among the involved brain areas, ACE2 has been found to be highly expressed in the regions involved in regulating cardiovascular functions, such as the subcortical organ, paraventricular nucleus, nucleus of the solitary tract, and rostral ventrolateral medulla [13].

Once SARS-CoV-2 infects the CNS, the effects are unpredictable, ranging from the absence of symptoms to severe clinical pictures (i.e., encephalitis), through mild manifestations (such as anosmia and ageusia). However, to what extent the CNS symptoms can be attributed to direct SARS-CoV-2 invasion or are fostered by secondary factors, either host-related or virus-induced, is not completely understood. The study of anosmia and hypogeusia in COVID-19 patients revealed that these symptoms are often associated with the female sex and are not associated with nasal obstruction, rhinorrhea, epistaxis, or nasal or head trauma.

The real incidence of these symptoms is unknown; however, it is likely to be highly underestimated, particularly from the first phases of the pandemic outbreak [14,15,16]. Anosmia and hypogeusia appear to be early symptoms of COVID-19 in selected patients, and they may occur before respiratory symptoms [17]. However, it is not clear whether these symptoms represent an unusual form of encephalitis or are due to the encephalopathy. Olfactory bulb MR imaging is useful for evaluating patients with anosmia/hyposmia, as it allows a deep examination of the brain areas involved.

Previous studies reported a decreased volume of the olfactory bulb in patients with post-infectious olfactory loss. This decrease might be related to the occurring symptom severity and olfactory loss duration. The gustatory and olfactory tracts often present hyperinflammatory signs that can be revealed by MR, as shown in Figure 1. In many cases, objective assessment of the olfactory dysfunction is lacking in the reports, which was underlined as a study limitation by several authors [18,19,20].

The increased signal intensity of the olfactory bulbs was not always present or, in some cases, was a pure expression of olfactory bulb damage [7]. The olfactory dysfunction may be due to injury to the olfactory supporting cells, or it could be related to direct damage to the olfactory nerves and retrograde invasion of the olfactory tracts [21]. We underline how the damage was still evident almost one month after the onset of the symptoms.

This case described a long neurological COVID experience: the long-lasting persistence of ageusia and anosmia, even after the infection, suggests persistent neuronal damage led by the SARS-CoV-2 infection. Neurological persistent damage is one of many collateral symptoms post-COVID-19 expression or also called long COVID. In many cases, dyspnea fatigue or anxiety still remains in hospitalized patients and in not hospitalized patients with less severe acute COVID-19 disease [22,23]. This study underlines the necessity and the urgency to further follow up with COVID-19 patients.

### 3.2. Case Report 2

A 55-year-old female was hospitalized for COVID-19 at the end of April 2020 and was admitted to the ICU for severe acute respiratory syndrome. The patient was treated with two doses of tocilizumab (8 mg/kg each) in the first 24 h and low molecular weight heparin (LMWH) at a prophylactic dose (4000 IU daily). She recovered respiratory function in 10 days, with a positive and effective return to spontaneous breathing. Neurological symptoms and delirium episodes were not present.

A search for coagulopathy was performed, and we found that the fibrinogen was compatible with control values during hospitalization. None of the following values were significantly altered: D-dimer, fibrinogen, prothrombin time (PT), and activated partial thromboplastin time (aPTT). All clinical parameters improved, respiratory function increased, and the serum IL-6 level was 123. 50 U/L.

**Figure 2:**
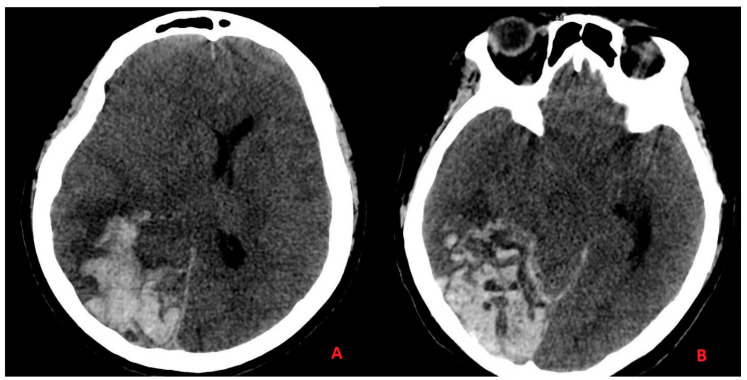
Acute ischemia with hemorrhagic infarction in the absence of any vascular malformation with acute quote of cerebral edema showed on CT head scan images. In panel (**A**), we can see the hemorrhagic infarction of the parietal and temporal lobes, with edema on the white matter of the antero-lateral portion of the temporal lobe with added “mass effect” on the adjacent structures. In panel (**B**), we can see the hemorrhagic infarction of the occipital and temporal lobes, and the subarachnoid hemorrhage on the cerebellar tentorium margin.

One day before the ICU planned discharge, the patient presented a sudden loss of consciousness with mydriasis and coma. Respiratory function was assisted for a neurological condition, and brain computer tomography (CT) and computed tomography angiography (CTA) was performed. We discovered acute ischemia with wide hemorrhagic infarction in the absence of vascular malformation with the acute quote of cerebral edema (Figure 2).

A carotid Doppler excluded any obstructions (a transcranial Doppler may be more informative; however, a physician was not always available to perform it, there was no surgical indication, and a poor neurological outcome was predictable) [24,25]. After 48 h, the patient died from withdrawal of life-sustaining treatment following the prognostication of a poor neurological recovery early after resuscitation. It was not possible to perform a post-mortem autopsy.

### 3.3. Discussion Part II—Ischemic Stroke and Intracranial Hemorrhage

Authors suggested that thrombotic and bleeding complications occurring in COVID-19 patients may represent different clinical manifestations of the same coagulation disorder, called COVID-19-associated coagulopathy (which is deemed to be distinct from sepsis-induced coagulopathy and disseminated intravascular coagulation, despite sharing some founding features) [26]. COVID-19-associated coagulopathy (CAC) typically shows increased D-dimer and fibrinogen levels, together with initially minimal alterations in the PT and platelet count [27,28].

Interestingly, CAC also shares some features with thrombotic thrombocytopenic purpura (TTP). In particular, the von Willebrand factor levels are increased in both CAC and TTP, due to vascular injury or reduced a disintegrin and metalloproteinase with a thrombospondin type 1 motif, member 13 (ADAMTS-13) levels, respectively, suggesting partially overlapping pathogenesis. It is not surprising that some classical TTP manifestations, such as stroke, acute coronary syndrome, and microvascular/arteriolar thrombosis, can also be observed in patients suffering from COVID-19, albeit with a significantly low incidence [26].

Endothelial dysfunction and macrophage hyperactivation are thought to be the main determinants of a peculiar immunothrombosis, leading to the shift of the hemostatic equilibrium toward a procoagulant state for microthrombosis, as well as venous and arterial thrombosis [26,27,28,29]. In summary, COVID-19-endotheliitis could explain the systemic impaired microcirculatory function in different vascular beds and the related clinical complications [30]. Relevant to this, recent autoptic studies of COVID-19 patients have shown the presence of fibrin thrombi within distended small vessels and capillaries, along with extensive extracellular fibrin deposition [31].

Interleukin (IL)-6-stimulated megakaryocytes and activated platelets may concur with immunothrombosis, increasing the risk of arterial thromboembolism [32]. As described by Fisicaro et al., vascular-related and infection-related secondary inflammatory tissue damage are caused by an abnormal immune response. However, it remains unclear whether these findings are the effect of a direct viral pathology or rather reflect a non-specific consequence of cardiovascular and pulmonary disease on the brain [33].

In our case, it was not possible to perform an autopsy. This cerebrovascular event was not expected, especially considering the risk factor, laboratory exams, and the low inflammatory levels expressed by the first cytokine level. The possible effects of treatment with Il-6 inhibitor are not clear [34,35]. The incidence of ischemic stroke in COVID-19 patients is approximately 1.5%, while it is typically rare in acute pneumonia or acute distress respiratory syndrome (ARDS) due to other causes.

Compared to cases who experienced a stroke without the infection, patients suffering from COVID-19 and stroke were younger and showed a higher frequency of large vessel occlusion as well as a higher in-hospital mortality rate [36]. Although ischemic stroke is more frequently observed in elderly COVID-19 cases, increasing the risk of cardiovascular complications [37], patients of all ages can be susceptible to large-vessel strokes [38]. Thrombotic complications predominate in COVID-19; however, bleeding (including intracranial hemorrhages) represents another relevant cause of morbidity and mortality ranging from 2% to 5.6% [39].

The close interplay between hyper-inflammation and hemostasis abnormalities inspired the use of heparin, whose therapeutic efficacy relies on both anti-inflammatory and anticoagulant properties. However, it is not possible to determine the risks and benefits of prophylactic anticoagulants for hospitalized people with COVID-19. Case report 2 illustrates that the widespread use of anticoagulant or anti-platelets drugs may result in an increased risk of severe hemorrhages.

It appears that the ischemic stroke was complicated by a hemorrhagic infarction, possibly fostered by the prophylactic use of heparin [40,41,42]. Similarly, another study reported a case of fatal intracranial bleeding in a patient affected by COVID-19 who was treated with heparin [27]. Another case series demonstrated that 6 of 11 COVID-19 patients presenting intracranial hemorrhage were on treatment with either antiplatelet agents or anti-coagulants [43].

## 4. Discussion

### Other Considerations and Neurological Aspects

As described by Kremer et al., the most frequent brain MR parenchymal signal abnormalities associated with severe acute respiratory syndrome are located in the medial temporal lobe nonconfluent white matter. It has been possible to observe multifocal white matter hyperintense lesions in association with hemorrhagic lesions in 30% of cases, and intracerebral hemorrhagic lesions in patients with a more severe clinical presentation were described in 54% of cases.

In addition to cerebrovascular lesions, Chougar et al. also reported perfusion abnormalities, cytotoxic lesions of the corpus callosum, two white matter-enhancing lesions, and basal ganglia abnormalities [44,45]. In cases with anosmia present, a high percentage of olfactory bulb degeneration was often associated with signal hyperintensity [7]. The reported cases described part of the common neurological manifestations of COVID-19, which may be characterized by headache, altered mental status, anosmia–ageusia, and, in the worst cases, encephalitis, stroke, and intracranial hemorrhage [46].

Thus, the exact pathogenetic dynamics underlying CNS damage remain unclear, and the manifestations are extensive [47]. The incidence of encephalopathy among COVID-19 patients can be attributed to many factors, such as medications, metabolic derangements. or sepsis, particularly in the case of exuberant cytokine production [48,49]. A sporadic neurological manifestation of COVID-19 involves acute myelitis, involving acute weakness of the lower limbs, urinary and bowel incontinence, and rapidly progressing to flaccid lower extremity paralysis and paresthesia numbness below T10 [50].

As reported by Kofitis et al. [51], delirium represents another neurological manifestation occurring in severe COVID-19 patients. Delirium could be a direct sign of CNS invasion, led by inflammatory mediators, or could be related to organ system failure, sedative strategies, and prolonged mechanical ventilation. The lack of positive environmental factors and the length of hospitalization in ICUs could increase delirium development [52].

The Confusion Assessment Method for the ICU score measurement remains a gold standard as an important tool to detect delirium as the first signal of neurological impairment [53,54]. The patients’ isolation, together with extended time away from family and the loved ones, could represent a source of delirium that healthcare professionals must address [55]. Thus, in-person visit or virtual connections via electronic devices (e.g., cell phone, tablet, or laptop) with family and friends could represents a valid strategy to avoid disorientation [56]. Based on what was observed from the cases presented in this study, important considerations can be made.

First, although specific neurological biomarkers of neurological involvement have not yet been identified, IL-6 and IL-10, together with the coagulation parameters, platelet count, and serum ferritin, represent important values to monitor. The NMDA testing should be performed in case of suspected encephalitis to facilitate the diagnosis [57,58,59].

Second, although a safe neuroprotection strategy is not available, Xu et al. suggested a possible treatment with vitamin D as a potential prophylactic, immunoregulatory, and neuroprotective treatment without side effects but further studies are necessary [60]. The role of sedative agents, such as halogenated vapor, could help to treat COVID-19-related acute respiratory distress syndrome and could exert some neuroprotective effects on the cerebral system [61,62].

Third, because symptomatic seizures can occur in patients suffering from COVID-19, EEG control should be adopted routinely in the case of severely sedated patients during the first 24 h of sedation and as weekly regular control in all prolonged durations of hospitalization [63].

Finally, as many asymptomatic carriers of SARS-CoV-2 present reductions in smell and/or taste, and this impairment is one of the earliest symptoms of COVID-19, olfactory/gustatory deficits could be used as a valuable, fast, and cheap screening tool for a preliminary diagnosis. The results of the screening could be implemented together with gene- or protein-based tests for viral particles.

## 5. Study Limitations

Case report 2 occurred during the first pandemic wave in Italy when hospitals were under high pressure and clinical practice faced increasing difficulties. Only a few centers were authorized for autopsy; in our case, this was not possible.

## Data Availability

Data are available upon request to the author.
